# An empirical strategy to detect bacterial transcript structure from directional RNA-seq transcriptome data

**DOI:** 10.1186/s12864-015-1555-8

**Published:** 2015-05-07

**Authors:** Yejun Wang, Keith D MacKenzie, Aaron P White

**Affiliations:** Vaccine and Infectious Disease Organization-International Vaccine Centre, University of Saskatchewan, Saskatoon, SK Canada; Department of Microbiology and Immunology, University of Saskatchewan, Saskatoon, SK Canada

## Abstract

**Background:**

As sequencing costs are being lowered continuously, RNA-seq has gradually been adopted as the first choice for comparative transcriptome studies with bacteria. Unlike microarrays, RNA-seq can directly detect cDNA derived from mRNA transcripts at a single nucleotide resolution. Not only does this allow researchers to determine the absolute expression level of genes, but it also conveys information about transcript structure. Few automatic software tools have yet been established to investigate large-scale RNA-seq data for bacterial transcript structure analysis.

**Results:**

In this study, 54 directional RNA-seq libraries from *Salmonella* serovar Typhimurium (*S.* Typhimurium) 14028s were examined for potential relationships between read mapping patterns and transcript structure. We developed an empirical method, combined with statistical tests, to automatically detect key transcript features, including transcriptional start sites (TSSs), transcriptional termination sites (TTSs) and operon organization. Using our method, we obtained 2,764 TSSs and 1,467 TTSs for 1331 and 844 different genes, respectively. Identification of TSSs facilitated further discrimination of 215 putative sigma 38 regulons and 863 potential sigma 70 regulons. Combining the TSSs and TTSs with intergenic distance and co-expression information, we comprehensively annotated the operon organization in *S.* Typhimurium 14028s.

**Conclusions:**

Our results show that directional RNA-seq can be used to detect transcriptional borders at an acceptable resolution of ±10-20 nucleotides. Technical limitations of the RNA-seq procedure may prevent single nucleotide resolution. The automatic transcript border detection methods, statistical models and operon organization pipeline that we have described could be widely applied to RNA-seq studies in other bacteria. Furthermore, the TSSs, TTSs, operons, promoters and unstranslated regions that we have defined for *S*. Typhimurium 14028s may constitute valuable resources that can be used for comparative analyses with other *Salmonella* serotypes.

**Electronic supplementary material:**

The online version of this article (doi:10.1186/s12864-015-1555-8) contains supplementary material, which is available to authorized users.

## Background

Lowered sequencing costs combined with dramatic increases in data output have greatly accelerated bacterial RNA-seq based transcriptome studies. RNA-seq can not only determine the absolute gene expression levels with lower variation compared to microarray technology, but can also be used to find new genes and resolve the structure of transcripts [1,2]. There are many tools for read mapping, gene expression normalization and comparison, most of which were originally designed for eukaryotic organisms but also useful for bacteria, e.g., Bowtie, BWA, edgeR, etc. [3-5]. For bacterial-specific transcript structure analysis from RNA-seq data, such as transcriptional start site (TSS) and transcriptional termination site (TTS) detection, operon identification, and small RNA identification, however, few studies have been performed to determine the technical feasibility, and fewer software tools have been developed [6,7].

Though most encoding genes and many non-coding RNAs have been identified for a large variety of bacteria, the gene structure and transcriptional unit organization are not clear. Understanding bacterial transcript structure is important for systems biology studies. The identification of TSSs and TTSs can help to define the downstream or upstream untranslated regions (5′- or 3′-UTRs, respectively), which often contain trans-acting regulatory DNA elements. In addition, after a TSS is determined for a transcriptional unit, the promoter can be delineated, which gives clues about gene regulation [8,9]. Genes that are transcribed in a single operon often have similar or coordinated functions, and participate in related pathways or biological processes [10,11]. Therefore, operon organization analysis can aid in identifying the function of unknown genes. Despite its importance, the transcript structure has only been determined in detail for a few bacterial strains [12,13], while for many others, including *Salmonella enterica*, the transcript organization has been resolved for only select groups of genes [14]. Recent studies have also revealed dynamic TSS and operon patterns in the same strains under different growth conditions, demonstrating the increased complexity of transcript structure analysis [15,16].

Traditionally, identification of TSSs and TTSs was heavily based on experimental strategies, such as 5′ or 3′-RACE (Rapid-Amplification of cDNA Ends), primer extension, and S1 nuclease protection mapping assays [17-20]. These methods are highly accurate, but their efficiency is low and cannot keep pace with the urgent need for a systems level understanding of bacterial gene regulatory networks. Alternatively, there are a number of machine-learning software tools established for TSS and TTS prediction purely based on features of known TSSs or TTSs [21-24]; however, the low prediction accuracy and reliability of these tools represent a severe problem. Moreover, most of the software tools were developed based on the features of *E. coli* and other model microorganisms, which may not reflect general properties for other bacterial TSSs and TTSs. To identify operons, the intergenic distances and co-expression coefficients are two major factors. However, it can sometimes be difficult to determine co-regulation between two adjacent genes in the same operon or between two adjacent operons. It is also not easy to identify alternative TSSs or TTSs within multiple-gene operons that are known to generate alternative transcripts [25].

Some groups have attempted to use RNA-seq to analyze TSSs in bacteria [14,26-31]. RNA-seq based TSS identification appeared more efficient than traditional experimental strategies and more precise than strictly bioinformatic methods. Most of these studies adopted the differential RNA-seq (dRNA-seq) method that enriches for primary mRNAs and degrades processing mRNAs [14,26,27,29-31]. In these studies, a large number of TSSs were identified, but the TTSs and operon organization could not be determined. Moreover, the TSSs were often identified manually or semi-automatically, the standards were varied and subjective, and no statistical reliability scores were given for the results.

Most RNA-seq experiments are performed to achieve the goal of comparative analysis of gene expression. Strand-specific (or directional) RNA-seq is often employed for these experiments and the resulting data represent an untapped resource for bacterial transcript structure analysis. However, several problems remain to be solved before this information can be extracted. How are the mapped cDNA reads distributed along bacterial transcripts? How can transcript structure be resolved according to these mapping patterns? How accurate and reliable are the results? If the identification of structural features is not precise, are the errors caused by the RNA-seq technique itself or by the analytical methods employed? This study was designed to address these questions. We sequenced multiple *Salmonella* cDNA libraries, observed the distribution of mapped reads along transcripts, proposed an empirical TSS and TTS detection method along with appropriate statistical tests, and compared the performance with a classical Poisson distribution based method. The methods developed in this research can be widely used to automatically detect bacterial TSSs, TTSs and operon organization from directional RNA-seq data. In addition, we provide comprehensive gene transcript structure annotation for the 14028s strain of *Salmonella* serovar Typhimurium, an important human and animal pathogen.

## Results

### Extraction of TSSs and TTSs based on real read mapping patterns

**Figure 1:**
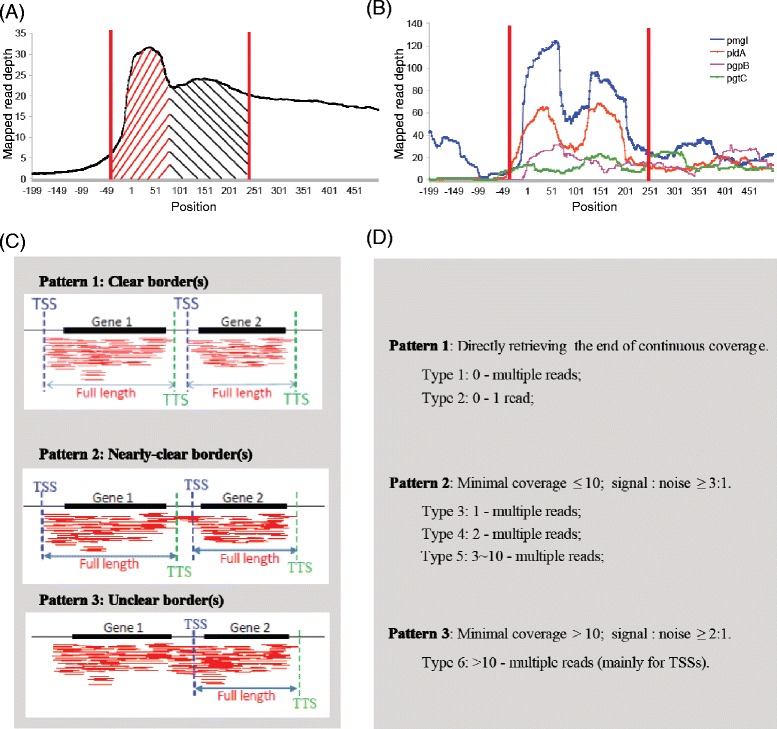
Read distribution, mapping patterns, and TSS/TTS extraction strategies for *S.* Typhimurium 14028s RNAseq analysis. **(A** and **B)** Average depth of mapped reads at different positions of encoding and flanking regions. Shaded regions in red and black indicated two theoretical peaks of high read enrichment **(A)** versus the actual pattern of mapped reads in individual genes **(B)**. The read-enriched region is indicated between the two red vertical lines. The horizontal axis represents the position along a gene, with the first nucleotide of the start codon as ‘0’ and the order of upstream positions being negative. **(C)** Schematic of two adjacent genes displaying different read-mapping patterns observed in our RNAseq experiment. **(D)** Description of Patterns 1, 2 and 3, sub-divided into Types 1–6. For each Type, the first number listed refers to the number of reads mapped between the TTS of Gene 1 and the TSS of Gene 2 and the second number refers to the number of reads mapped to the Gene 2 transcript. The Signal to noise ratio refers to the second number divided by the first number.

We developed an empirical method, based on the biased distribution of reads at the 5′ end of transcripts, to extract transcript structure information from standard bacterial RNA-seq data. Within our RNAseq dataset, we observed that known transcriptional units, either generally or individually, were enriched for their read coverage at 5′-ends as well as upstream regions (Figure 1A and B). This agrees with Raghavan et al. [32], who first reported an increased distribution of cDNA reads mapping to the upstream region of genes. A similar biased distribution of reads has also been observed for eukaryotic mRNAs [33]. A biased distribution is problematic because the existing methods for extracting transcript information from bacterial RNAseq data are based on Poisson distribution models, which assume a hypothetically even distribution across an entire transcript [7,29]. We reasoned that if cDNA reads are not uniformly distributed across transcripts that it would limit the effectiveness of Poisson distribution-based methods. The new empirical method that we propose is based on the actual read mapping patterns at gene borders and is independent from any theoretical distribution model or the hypothesis of ‘even distribution along the transcript’.

The criteria and TSS/TTS retrieving strategies for our empirical method are outlined in Figures 1C and D. If the borders between transcripts were clear or nearly clear (Figure 1C, Pattern 1 and Pattern 2), it was easy to distinguish the TSSs and TTSs. However, sometimes the borders were unclear and it was difficult to define the transcript structure (Figure 1C, Pattern 3). These regions could reflect sub-operons, where one gene or sequential genes within a known long operon can be transcribed as independent transcriptional units. Based on the property of biased read distribution, we detected TSSs in some of these areas (Figure 1C and D, Pattern 3), factoring in the signal to noise ratio (i.e., # reads mapping to 5′ region of Gene 2/# reads mapping to region downstream of Gene 1). The TSSs and TTSs obtained from Patterns 1, 2 and 3 were further classified into 6 Types (Figure 1D). Type 1 TSSs and TTSs had multiple (≥2) read coverage at the TSSs/TTSs junctions and no reads upstream of the TSSs or downstream of the TTSs. Type 2 was classified as a situation with only single read coverage at the TSS/TTS junction and no reads upstream of the TSSs or downstream of the TTSs. Type 2 cases, therefore, were subject to more noise and had a greater chance that this was due to random sequence. Types 3, 4, and 5 belong to Pattern 2, but differed based on noise strength: for Type 3, 4 and 5, there was single, double, and 3–10 read coverage upstream of the TSSs or downstream of the TTSs. For Type 6, the read coverage was >10 at positions adjacent to the TSS/TTS, which could represent part of other transcripts, as described above.

In our preliminary analysis, the resolution of Type 1 TSSs/TTSs was strikingly different from that of Types 2–6; the number of Type 2, 3, 4, and 5 TSS/TTSs was decreasing but still high, while that of Type 6 was quite small, and therefore no additional types were defined. We tested different combinations of minimal coverage and signal-to-noise ratios (data not shown), but the final parameters were selected based on the best sensitivity, ensuring that most of the real TSSs and TTSs were captured (Figure 1D).

### Consistency of the TSSs and TTSs detected from directional RNA-seq libraries

Since few TSSs/TTSs have been validated for most bacterial strains, an alternative strategy for evaluating the reliability of prediction results is to test the overlap of TSSs/TTSs extracted from different RNAseq libraries. It is assumed that the most reliable transcript features will be consistent between libraries. The TSSs/TTSs of known encoding genes of *S*. Typhimurium 14028s identified from a single RNAseq library were summarized and then compared with those from replicated libraries (Table 1). On average, from a single library, TSSs were captured from ~17% of the protein-encoding genes in the 14028s genome, with Type 1, Type 2, Types 3–5 and Type 6 (Figure 1C) representing 23%, 67%, 7% and 3% of the total, respectively. The remaining ~83% of genes without captured TSSs were either lowly expressed or not expressed, expressed as part of a long operon, or failed to satisfy the criteria for TSS analysis, such as read coverage or signal to noise ratio.

**Table 1:** **Summary of TSSs and TTSs identified from a single RNA-seq library**

**Type**	**TSS**		**TTS**	
	**Number (%)** ^**1**^	**Consistency** ^**3**^	**Number (%)** ^**1**^	**Consistency** ^**3**^
Type 1	206 ± 88 (22.9 ± 4.4)	88.8 ± 8.6%	85 ± 47 (10.6 ± 2.8)	70.2 ± 16.4%
Type 2	610 ± 323 (66.9 ± 3.6)	78.1 ± 8.6%	656 ± 370 (81.0 ± 3.6)	58.2 ± 15.6%
Type 3-5	68 ± 28 (7.2 ± 2.4)	91.4 ± 10.5%	46 ± 33 (5.7 ± 1.5)	85.0 ± 18.3%
Type 6	30 ± 20 (3.0 ± 2.4)	96.9 ± 9.8%	24 ± 30 (2.8 ± 1.9)	96.3% ± 12.8%
Total	914 ± 493 (17.2 ± 9.3)^2^	82.0 ± 7.7%	812 ± 470 (15.3 ± 8.8)^2^	61.3 ± 14.4%

Note: ^1^The 95% confidence limits (mean ± 1.96SD) of the number or percentages of TSSs/TTSs identified from a single library were represented. For Type 1, 2, 3–5, and 6, the percentages of respective type(s) from total number of TSSs/TTSs are given.

^2^The percentages of *S*. Typhimurium 14028s chromosome-encoding genes represented by the total identified TSSs/TTSs were given.

^3^The percentages of TSSs/TTSs (individual types or total) identified by both two independent libraries within ±50 nt errors among the total number of TSSs/TTSs identified by both libraries were given as 95% confidence limits.

**Figure 2:**
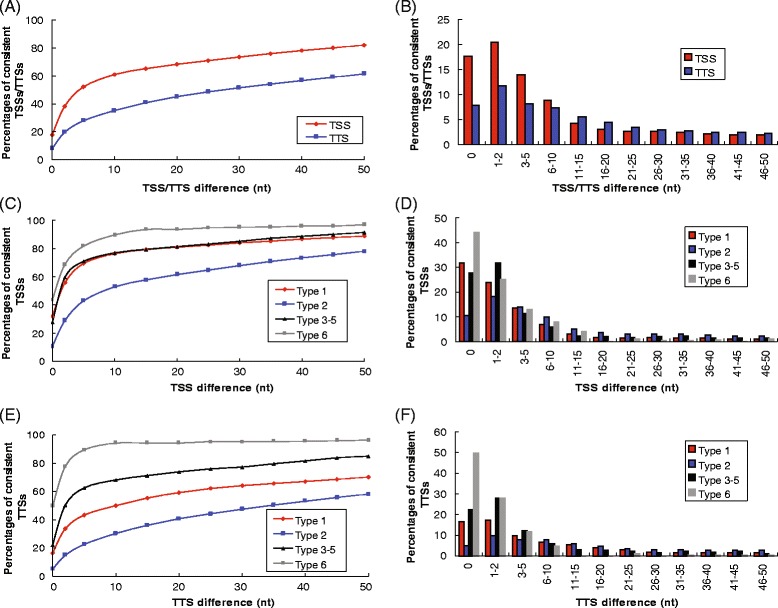
Consistency of TSSs and TTSs detected from repeated libraries. **(A)** The cumulative percentages of TSSs and TTSs consistently captured by two libraries within different levels of resolution (allowing for position differences). **(B)** The actual percentages of TSSs and TTSs consistently captured by repeated libraries with various position differences (0 = identical positions; 1–2 = 1–2 nucleotides apart; etc.). The cumulative percentages of library-consistent TSSs **(C)** and TTSs **(E)** of different types (as in Figure 1D) are plotted at different levels of resolution. The actual percentages of library-consistent TSSs **(D)** and TTSs **(F)** of different types with various position differences are shown.

A large number of TSSs and TTSs could be identified within two independent libraries when a certain level of position difference was allowed (Table 1; Figure 2A-B). Approximately 18% of the TSSs identified from both libraries were exactly the same, and 61% or 82% were within ±10-nt or ±50-nt difference, respectively (Figure 2A-B). For TTSs, the consistency between libraries was inferior to that of TSSs, with 8% being exactly the same, and 35% and 61% within ±10-nt and ±50-nt limits, respectively (Figure 2A-B). The lower consistency of TTS identification could be attributed to lower read coverage depth at TTSs.

Among the different types of TSSs and TTSs, Type 1, 3–5 and 6 were all highly consistent between different libraries, with 28-44%, 76-90% and 89-97% within 0 nt, ±10 nt and ±50 nt for TSSs, and 16-50%, 50-94% and 70-96% within 0 nt, ±10 nt and ±50 nt for TTSs respectively (Table 1; Figure 2C-F). Type 2 TSSs and TTSs were the most abundant but showed the worst consistency between libraries, with 11%, 53% and 78% within 0 nt, ±10 nt and ±50 nt for TSSs, and 5%, 30% and 58% within 0 nt, ±10 nt and ±50 nt for TTSs respectively.

Taken together, the reliability of TSSs/TTSs detected from a single directional RNA-seq library was low at a single-nucleotide resolution, but moderate to high for ±10-50 nucleotide resolutions. If a single library is used for detection of TSSs/TTSs, the Type 1 and Types 3–6 are more precise, but the accuracy for Type 2 is much lower; therefore, application of a ±50 nt difference is suggested as the resolution confidence limit (82% for TSSs and 61% for TTSs).

### Enrichment and statistical refinement of TSSs and TTSs with multiple RNA-seq libraries

To increase the number of TSSs/TTSs identified from directional RNA-seq libraries, a strategy of combining multiple libraries was adopted. Statistical models were also developed to improve the reliability and precision of TSSs/TTSs.

**Figure 3:**
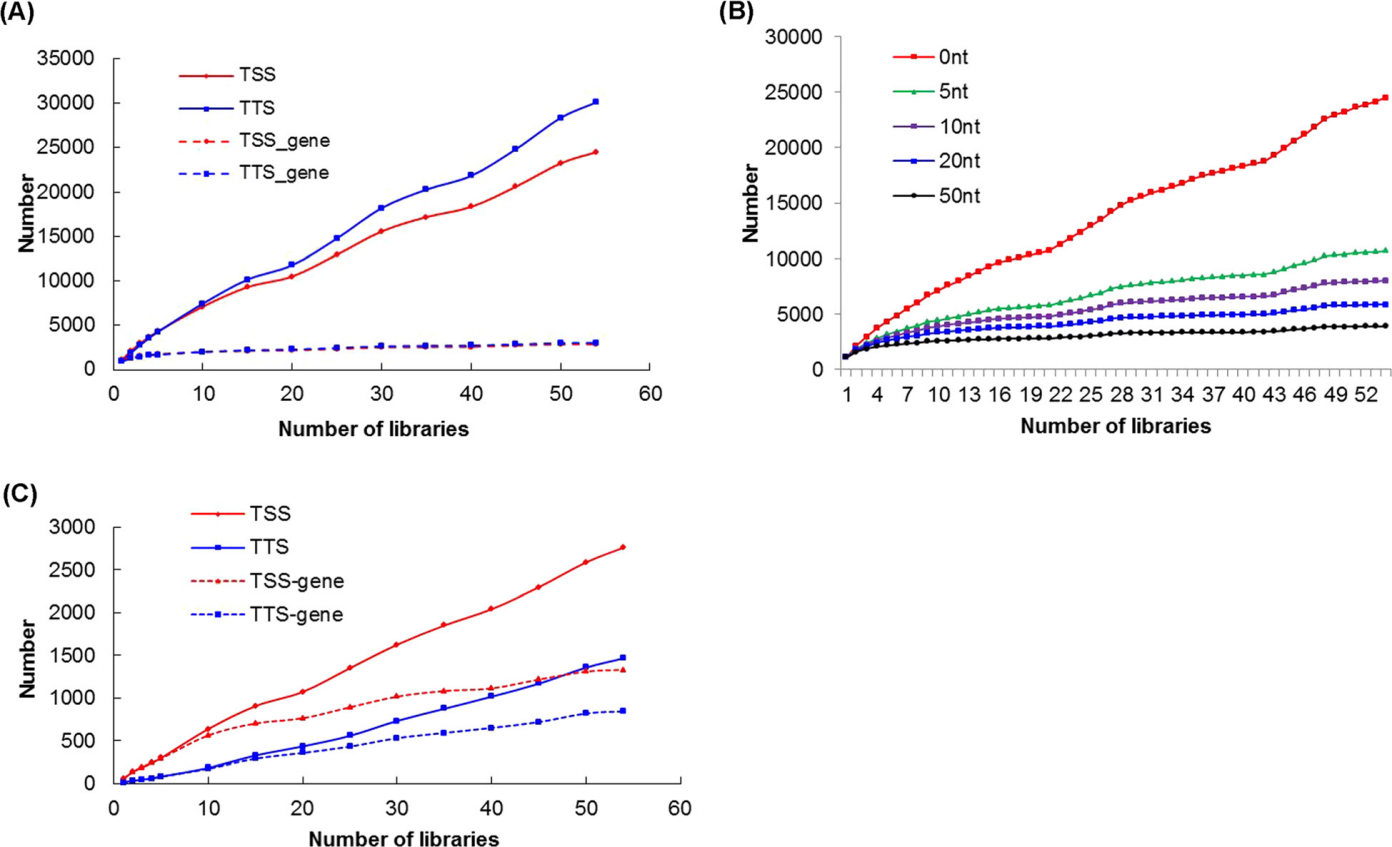
Enrichment of TSSs and TTSs with multiple RNA-seq libraries and the application of statistical tests. **(A)** Enrichment of detected TSSs/TTSs and corresponding genes as multiple RNA-seq libraries were compared. If transcript features (TSS/TTS) were ≥1 nucleotide apart in different libraries, they were counted as new features. **(B)** Enrichment of detected TSSs/TTSs at different resolution (allowing for position differences) with multiple libraries. **(C)** Enrichment of statistically significant detected TSSs/TTSs and corresponding genes with multiple libraries.

The total number of TSSs/TTSs identified increased linearly as more libraries were sequenced (Figure 3A). The number of genes whose TSSs/TTSs were detected was also increased, but with a much smaller slope, indicating that multiple TSSs/TTSs were identified for each gene. Most of these are not alternative TSSs/TTSs but rather reflect the difficulty of achieving single-nucleotide resolution; variability was decreased as we allowed more variance in the starting nucleotide position (Figure 3B for TSSs; similar curves were observed for TTSs (data not shown)).

Two different statistical models were developed to calculate the probability that each TSS/TTS was reliable. One method was based on the total read coverage and the other method was based on repeated detection in replicate RNAseq libraries (see description in Methods). With these two tests, among the 24,516 TSSs and 30,118 TTSs with single-nucleotide resolution detected from total 54 libraries, only 2764 and 1467 were significantly reliable, representing 1331 and 844 different genes, respectively (Table 2). The number of significantly reliable TSSs/TTSs and the non-redundant genes that they represent was also increased when more libraries were included (Figure 3C).

**Table 2:** ***Salmonella*** **Typhimurium 14028s TSSs and TTSs identified from 54 RNA-seq libraries**

	**Transcript Feature** ^**1**^	**Gene** ^**2**^
TSS		
Total number	24,516	2,888
Significant number^3^	2,764	1,331
TTS		
Total number	30,118	1,467
Significant number^3^	3,026	844

^1^Total number of transcript features identified; for each gene, different TSSs or TTSs could be identified from different RNA-seq libraries.

^2^The number of *S.* Typhimurium 14028s protein-encoding genes whose TSSs or TTSs were identified.

^3^Binomial tests based on read-depth and reproducibility between libraries were performed to find out the TSSs or TTSs that were statistically reliable. The significance level was set as FDR < 0.05 for either test.

**Figure 4:**
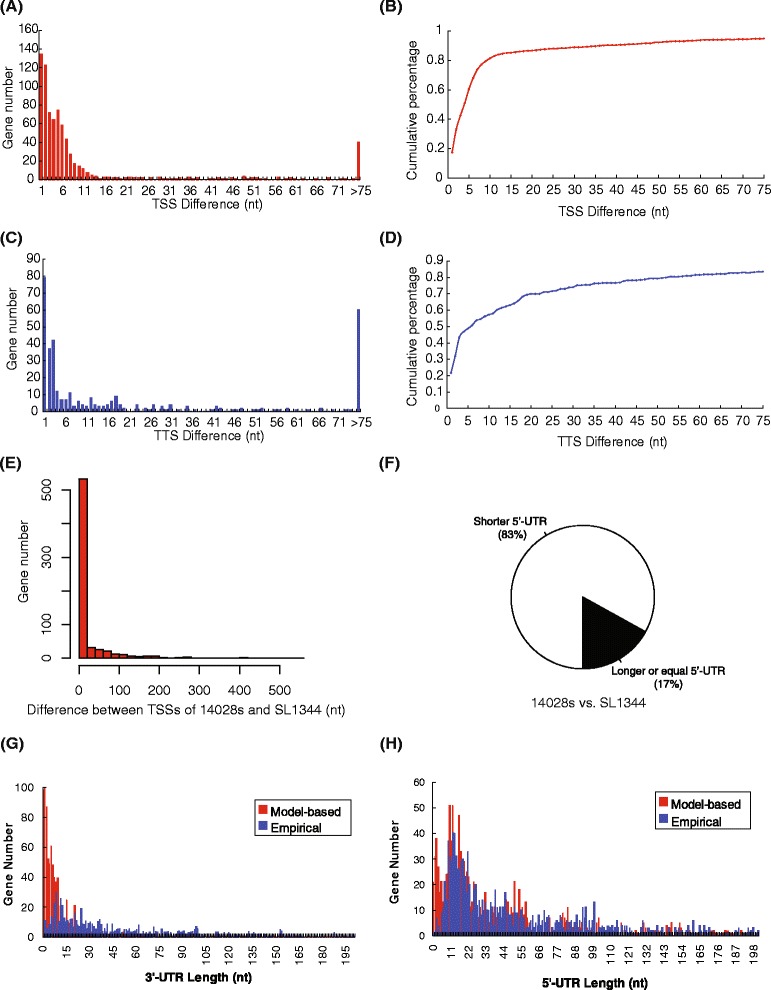
Accuracy of TSSs and TTSs detected from *S.* Typhimurium 14028s directional RNA-seq data. Our empirical method was combined with statistical tests to yield **(A)** the actual and **(B)** cumulative distribution of position differences between TSSs detected for individual genes. This data is matched with Figure 3C. The actual **(C)** and cumulative **(D)** distribution of position differences between TTSs detected for individual genes. **(E)** The consistency of detected TSSs between *S.* Typhimurium 14028s and SL1344 strains. **(F)** Comparison of 5′-UTR length of 14028s genes and SL1344 genes based on the TSSs detected in this study and from Kröger data [14], respectively. ‘Shorter’ or ‘longer’ meant that the 5′-UTRs were shorter or longer in 14028s than in SL1344, respectively. Any nucleotide position influences caused by inter-strain genetic differences were excluded from this analysis. Comparison of 3′-UTR length **(G)** and 5′-UTR length **(H)** between the Poisson distribution model-based method and our empirical method.

Even with the inclusion of statistical tests, there were still multiple TSSs for 57.8% (769/1331) and TTSs for 43.1% (364/844) of the represented genes. A small percentage of the TSSs/TTSs detected for single genes had relatively large position differences and could possibly represent alternative transcripts. However, the majority of TSSs (81.4%) and TTSs (57.3%) only varied within ±10 nt (Figure 4A-B for TSSs; Figure 4C-D for TTSs). Taken together, the addition of statistical tests significantly increased the resolution and precision for detecting TSSs and TTSs.

Recently, Kröger et al. performed a series of dRNA-seq experiments to analyze the TSSs of *S*. Typhimurium SL1344 [14]. Although there was no suggestion that SL1344 and 14028s had the same TSSs, since these strains are phylogenetically close and belong to the same species and serovar, we reasoned that the TSSs could be same for orthologous genes. Therefore, the TSSs identified from 14028s in this research were compared with those of SL1344. In total, 1075 protein-encoding genes (1075/1110, 97%) from Kröger SL1344 TSS list have orthologous counterparts in 14028s. Among them, 678 (678/1075, 63%) were also identified with TSSs in 14028s in this research (Additional file 1). Nearly 70% (533/678) of the TSSs had only a ±20 nt difference between 14028s and SL1344 (Figure 4E; Additional file 1). The high consistency between the two sets of TSSs demonstrated the effectiveness of our empirical method. It should be pointed out that the TSSs detected in 14028s and SL1344 were seldom detected at exactly the same position, with the majority (~83%) of TSSs of SL1344 located upstream of 14028s TSSs (Figure 4F). This indicated the possible technical disadvantage of directional RNA-seq compared with dRNA-seq to cover the 5′-ends of primary transcripts (Figure 4 F).

The 3′-UTRs detected with our mapping pattern-based empirical method were typically short, with 56%, 66% and 75% with length shorter than 50 nt, 75 nt, and 100 nt, respectively (Figure 4G). A single-parameter Poisson distribution-based model, such as used in [7], was also tested to extract the transcript borders from directional RNA-seq data. The 3′-UTRs based on the Poisson distribution-based model were shorter than those with our empirical method (Figure 4G). For 5′-UTRs, ~60% identified with our empirical method were between 15 and 100 nt length while the average length calculated from the distribution-based model also appeared shorter (Figure 4H). Taken together, these results demonstrated that the uneven distribution of mapped reads along a transcript could make the model-based border analysis inaccurate, and for these cases, an empirical strategy simply based on the mapping patterns could be a better choice.

**Figure 5:**
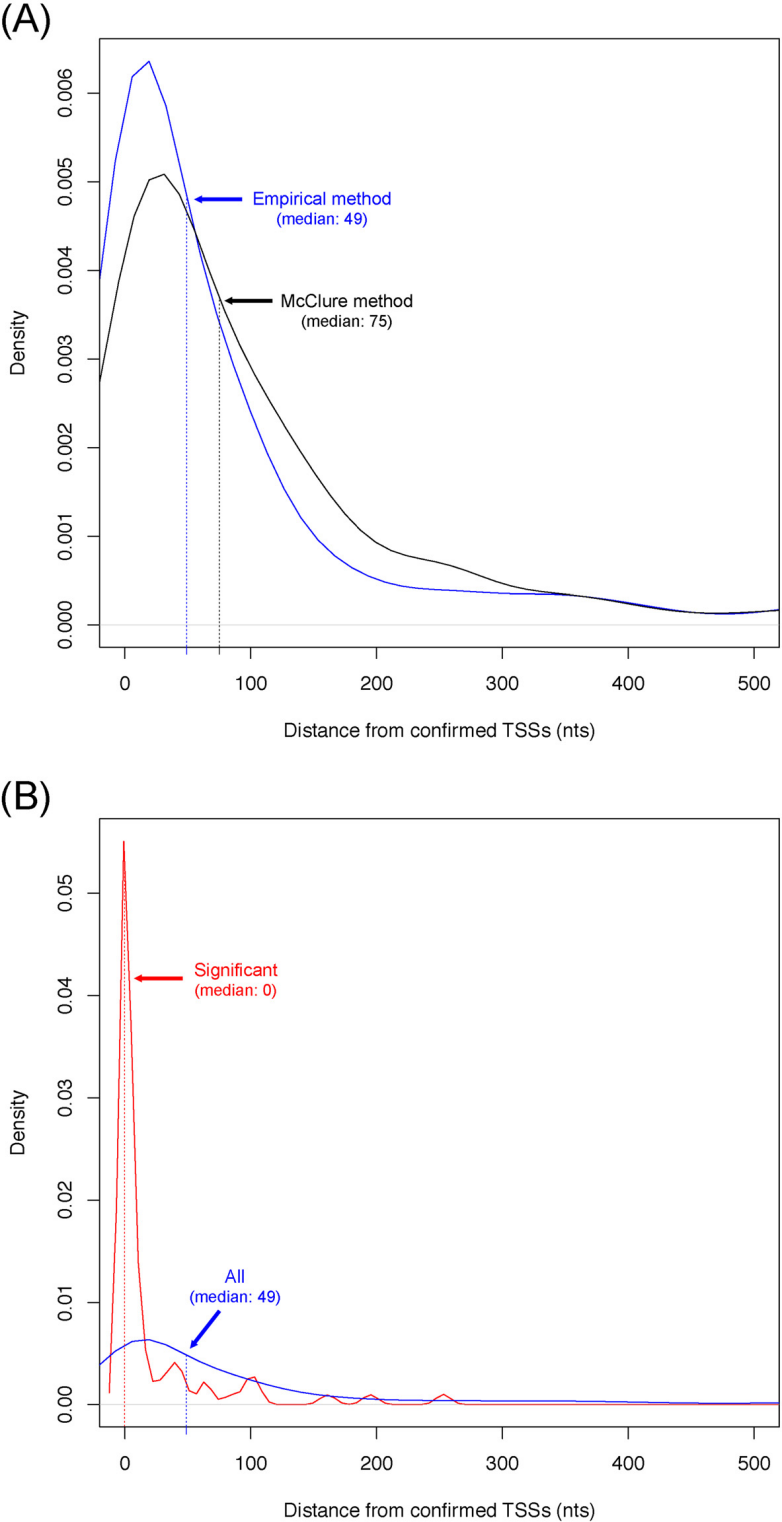
Performance comparison of tools on TSS prediction from *E. coli* GAII RNA-seq data. **(A)** Distance distribution between validated TSSs and those predicted with the empirical method (blue) and the distribution-based McClure method [7] (black). **(B)** The distance distribution between 70 TSSs predicted with the empirical method that had also been experimentally validated. The blue and red curves represent total and statistically significant TSSs respectively. In both **(A)** and **(B)**, the distance between predicted and validated TSSs and corresponding distribution density were shown on horizontal axis and vertical axis, respectively. The median distances were indicated beside each corresponding distribution curve.

To further confirm the general applicability of our empirical method on directional RNA-seq data of different species, three *E. coli* strain K-12 libraries were analyzed (see Methods). In total, 1387 non-redundant TSSs and 1399 TTSs were detected (Additional file 2). Among the 630 predicted TSSs that had been experimentally validated (http://regulondb.ccg.unam.mx/), the median distance between the predicted and validated ones was 49-nt, and 31% were with a distance within 10-nt (Additional file 2). Considering that these *E. coli* datasets represent the responses to three different stresses, and the potential for many unknown and alternative TSSs due to dynamic operon organization [6], the actual TSS prediction performance could be better. We also re-implemented McClure’s Poisson distribution-based method [7]. For the 630 common TSSs, the median distance between the predicted results and the validated TSSs was 75-nt, and only 16% of them were with a distance within 10-nt (Figure 5A). Since the number of libraries analyzed was small, only read number based statistical tests could be performed. 145 TSSs and 53 TTSs were statistically significant without multiple testing correction (*p* < 0.05), and only 85 TSSs and 22 TTSs were significant after correction (FDR < 0.05). 70 of the 145 significant TSSs had been experimentally verified (http://regulondb.ccg.unam.mx/); the median distance between the significant TSSs and the validated ones was 0, and 70% were within a distance of 10-nt (Figure 5B). Therefore, although our empirical method loses sensitivity by taking the proposed statistical tests, the precision of transcript border identification is highly increased. Taken together, the results demonstrated the applicability of our empirical method for transcript border analysis on directional RNA-seq data of various bacterial species, from different sequencing platforms, and with more or few replicates. The method also had improved performance when compared with a Poisson distribution-based method.

### Inventory of *S*. Typhimurium 14028s operons

**Figure 6:**
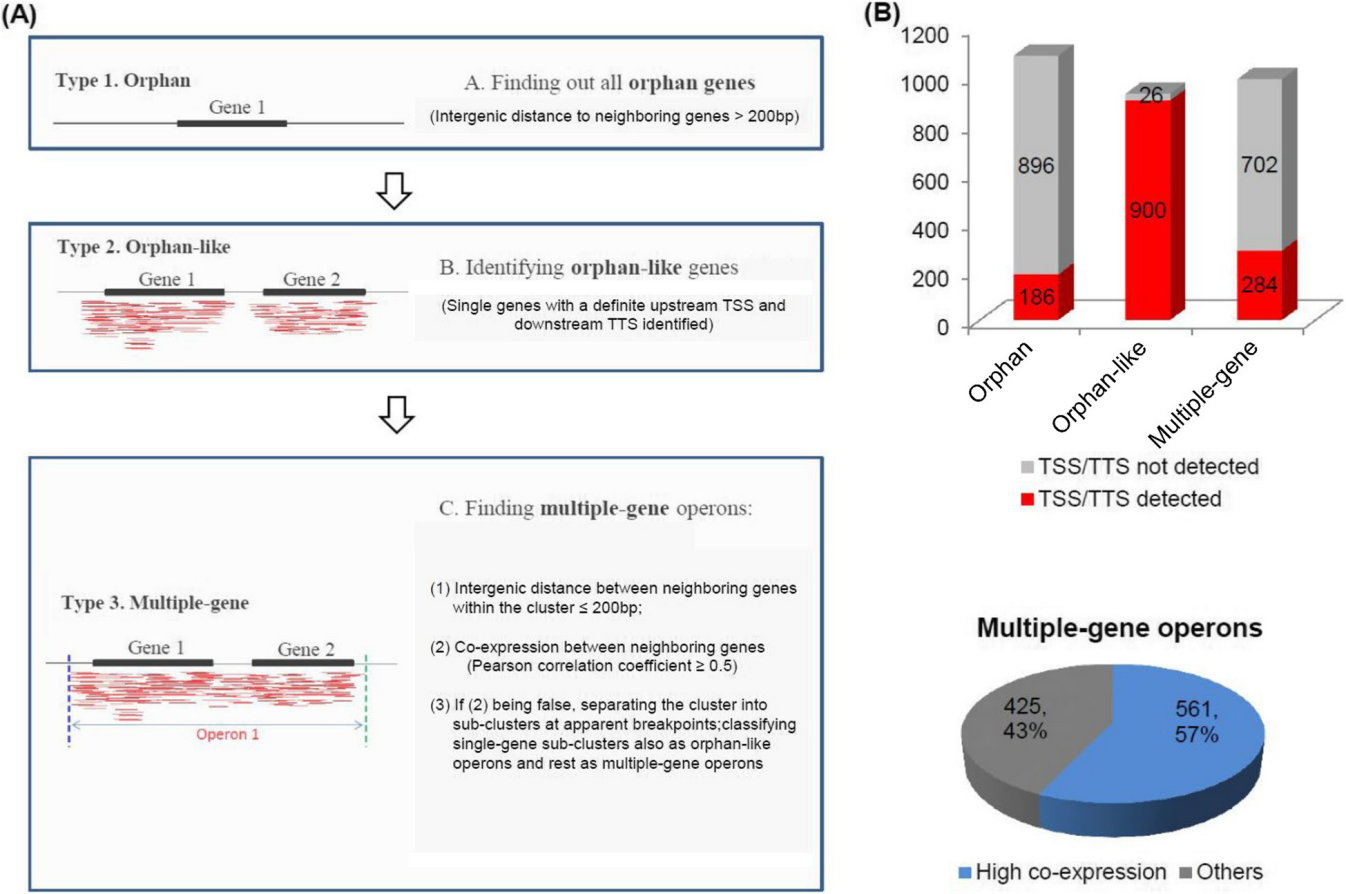
Pipeline for operon organization analysis and summary for *S*. Typhimurium 14028s. **(A)** Classification of operons. The parameters are described in more detail in the Methods. **(B)** Summary of *S*. Typhimurium 14028s operons divided into three categories and the proportion that had TSSs and/or TTSs detected. The pie chart below shows the co-expression of genes within the same multiple-gene operons. Operons with high co-expression are those with an average co-expression PCC > 0.5 between adjacent genes. Others refer to operons with an average co-expression PCC <0.5 between adjacent genes, or those with genes that were lowly expressed or not expressed under any condition.

We used a combination of TSS and TTS information, intergenic distance and co-expression coefficients between neighboring genes to screen for all possible operons in the *S*. Typhimurium 14028s genome. The operons were classified into Type 1 (orphan), representing single genes with long intergenic distances to neighboring genes, Type 2 (orphan-like), representing single genes with shorter distances to adjacent genes but having defined TSSs and TTSs, and Type 3 (multiple-gene), consisting of transcriptional units composed of multiple adjacent genes (Figure 6A). The cutoffs for intergenic distance and the co-expression coefficients are defined in the Methods.

The 5311 chromosome-encoding genes of *S.* Typhimurium 14028s were organized into 1082 orphan, 926 orphan-like and 986 multiple-gene operons (Additional file 3; Figure 6B). 186 orphans, 900 orphan-like operons and 284 multiple-gene operons were detected with TSSs and/or TTSs, and 561 multiple-gene operons were confirmed by high expression correlation coefficients between constituent genes (Additional file 3; Figure 6B). Based on the operon organization table, 694 hypothetical genes with unknown function were categorized into multiple-gene operons with validated co-expression with adjacent genes (Additional file 3). The proteins encoded by the genes within each of these operons should have related or coordinated function, participate in the same or related biological processes, or encode different functional components of some molecular machines, cellular components or functional complexes. The organization of these hypothetical genes into operons represents a starting point to investigate their cellular functions.

### Sequence features of *S*. Typhimurium 14028s TSSs, TTSs and promoter regions

**Figure 7:**
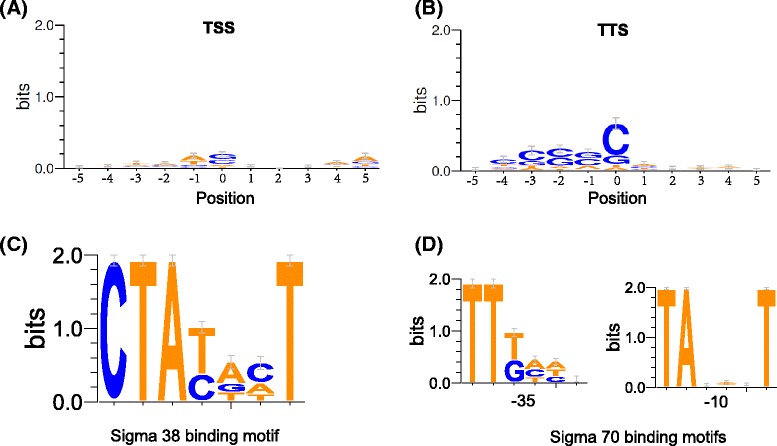
Position-specific nucleotide composition features of transcription border regions or promoters. Nucleotide composition at TSSs **(A)** or TTSs **(B)** predicted using the empirical method. The position ‘0’ represents the TSS or TTS, minus values represent upstream positions and positive values represent downstream positions. Nucleotide composition of the −10 region of putative sigma 38 binding sites **(C)** or the −35 and −10 regions of putative sigma 70 binding sites **(D)** identified in the *S.* Typhimurium 14028s chromosome.

We further analyzed the TSSs and TTSs to identify any potential nucleotide compositional bias at adjacent chromosomal positions. Previous studies showed an apparent preference of A/T at TSSs [14]. However, we did not observe any apparent nucleotide composition bias for TSSs (Figure 7A). This discrepancy could be attributed to incomplete mapping at the 5′ ends of many primary transcripts due to the technical limitations of directional RNA-seq. In contrast, for TTSs, the position 0 and 3 upstream neighboring positions (within-gene) all had an apparent G/C enrichment (Figure 7B). This feature was similar with Petersen and Krogh’s finding, indicating the possible importance of Rho-dependent transcriptional termination in *S*. Typhimurium 14028s [23].

Sigma 70 (RpoD) and sigma 38 (RpoS) are two principal RNA polymerase sigma factors in *Salmonella*. Sigma 70 plays a primary role during exponential growth, regulating the expression of a large number of genes that are essential for normal growth. In contrast, sigma 38 is highly expressed during stationary phase and is the central regulator of the general stress response [34]. Both sigma factors bind promoter regions of their corresponding regulons with similar but distinct sequence preferences [35,36]. According to respective binding patterns, the promoter regions of *S.* Typhimurium 14028s chromosomal genes, defined by their identified TSSs, were screened for possible sigma 70 and sigma 38 binding motifs. In total, 215 putative sigma 38 binding motifs (Figure 7C; Additional file 4) and 863 sigma 70 binding motifs (−10 box) were detected between the −35 to −1 positions (Figure 7D; Additional file 4). The detection of such a large number of putative sigma factor motifs further demonstrated the reliability of the TSSs detected in this research, given the acceptable resolution. The ‘-35 box’ of sigma 70 binding sites was often significantly degenerate, and therefore difficult to detect [14]. However, 172 genes were still discerned to contain both −35 and −10 sigma 70 binding boxes (Figure 7D; Additional file 4).

## Discussion

An apparent advantage of RNA-seq-based versus microarray-based transcriptome analysis is that RNA-seq can provide transcript structure information. However, until now, no direct analysis has been performed to examine whether directional RNA-seq data can be used for transcriptional border identification. It remains to be clarified what is the best resolution possible for bacterial TSSs and TTSs identified from directional RNA-seq data. In this research, we tried to answer this question. Currently, there are two main methods applied for transcript border analysis from bacterial RNA-seq data - manual annotation and distribution model based methods [7,14]. For well-presented patterns (e.g., TEX-treated RNA-seq data, see later in this paragraph) or particularly interesting genes, a manual strategy can generate the most accurate and useful results. However, manual analysis of thousands of transcripts per library is an unwieldy process that is not suitable for large-scale analysis. On the other hand, the use of distribution-based models to analyze standard, non-TEX-treated RNA-seq data is also difficult. Since the transcript borders are not clear, the inclusion of any subjective or inconsistent criteria would lead to unreliable results. This is precisely what we observed for a Poisson distribution-based model, which makes the assumption that reads are evenly distributed along the transcript. In contrast, we observed a biased distribution of reads along a transcript. Non-uniform read distribution has also been found in eukaryotic RNA-seq data, and could be related with the sampling preference of secondary sequencing technologies [33]. Alternatively, a large amount of immature mRNAs could also lead to the 5′-biased read number [32]. Based on this finding we proposed a new empirical method, which is independent of the theoretical distribution of reads along transcripts. A direct comparison of our empirical method with distribution model-based methods confirmed that our model achieved more accurate results. We determined that the best resolution of transcript borders retrieved from RNA-seq data was within ±10-20 nt difference from experimentally determined borders. The difficulty in reaching single-nucleotide resolution was due to limitations in the directional RNA-seq technique itself and not because of limitations with our method. Several groups have reported difficulty in capturing 5′ ends of the primary mRNAs with standard library preparations and directional sequencing strategies [14,27]. A terminator 5′-phosphate-dependent exonuclease (TEX)-treated library-generating strategy has been frequently adopted to enrich for primary mRNAs. Although the TEX method has resulted in accurately defining a large number of TSSs in many bacterial species, it is much more expensive than normal library preparations and it does not enrich for the 3′ ends of transcripts. Moreover, TEX has only been used for a limited number of studies in comparison to standard RNA-seq analysis, which has been used for numerous bacterial species. Therefore, the automatic TSS and TTS detection method that we have described in this research should have wide application for bacterial transcriptome studies. A resolution of ±10-20 nt is predicted to be sufficient for transcript unit recognition, promoter definition, motif finding, and the identification of new genes.

The resolution of TSSs and TTSs was improved as sequencing depth increased or more libraries were included in the analysis. For example, with 54 directional RNA-seq libraries, each averaging 20 M 75 bp reads with ~90% ribosome RNAs and transfer RNAs, we identified 2764 TSSs and 1467 TTSs for 1331 and 844 genes in *S*. typhimurium 14028s, respectively. The resolution appeared to reach a maximum threshold of ±10-nt when compared to TEX-generated TSSs [14]. Within this maximal resolution limit, there will always be a trade-off between sensitivity of TSS/TTS detection and resolution when the size and number of libraries are fixed. For example, using a single library of 20 M total reads with ~90% rRNAs/tRNAs, we could detect the TSSs for ~900 genes and TTSs for ~800 genes at an average resolution of ±50 nt. In general, there was a positive linear correlation between the number of TSSs/TTSs detected and the mRNA sequencing depth and/or the number of biological replicate libraries analyzed. Based on our observations, we suggest that statistical tests and the maximal resolution should be applied for TSS detection when >5 libraries are being analyzed, consisting of at least 10 M mRNA-mapping reads. When the number of libraries is smaller than 5 or the total number of mRNA-mapping reads is smaller than 10 M, we suggest that TSSs be detected with highest sensitivity, focusing on genes that have clear read-mapping borders (Figure 1C, Pattern 1). For TTSs, we suggest that the same criteria apply except with the high-resolution cutoff being 10 libraries and 20 M total mRNA-mapping reads. As noted above, both the sequencing depth and gene expression level can influence transcript border identification. To improve accuracy, in the default settings of our method, the genes with <70% whole coding region coverage were filtered out. Although transcript borders can still be identified with only a few libraries or low general sequencing depth (e.g., the *E. coli* TSSs and TTSs identified in Additional file 2), integration of more RNA-seq libraries under different stresses is still recommended, which will increase both the prediction resolution and the number of borders identified.

For *S*. Typhimurium 14028s, in addition to the 54 RNAseq libraries that we analyzed here, we are in the process of analyzing 12 other cDNA libraries. Together with the efforts being made by other research groups, we anticipate that more TSSs and TTSs will be delineated in the near future. As mentioned above, it will likely not be possible to reach a single-nucleotide resolution, but that should not influence the analysis of transcription unit organization, promoters, and even dynamic transcription structure under different conditions or stresses. The TSSs, TTSs and operons identified in this study represent the first version of a transcript structure profile in *S.* Typhimurium 14028s. As more and more directional RNA-seq data (or even TEX-treated RNA-seq data) are available, the transcription structure information will be updated by simply re-implementing the methods, pipelines and statistical models developed in this study. The methods can also be applied to RNAseq datasets obtained from other bacteria.

We did not identify any nucleotide preference at TSSs or adjacent positions, but did observe C/G enrichment at the TTS as well as the 3–4 adjacent positions upstream. The inconsistency of nucleotide composition bias at TSS between our results and previous observations [14] was most likely due to unmapped 5′ ends of primary mRNAs in our experiments. For C/G preference at TTS, it is consistent with previous observation on Rho-dependent transcription termination sites [23], implicating potential involvement of the Rho factor in transcription termination in *S.* Typhimurium. There remains a need to determine whether the 5–10 nt downstream of TTSs also showed an apparent nucleotide composition bias as shown in [23], and to further understand the contribution of Rho-dependent and Rho-independent transcription termination in *Salmonella*.

Finally, we detected putative sigma 38 binding motifs in the −10 region of promoters from 215 *S.* Typhimurium 14028 genes. Considering the slightly shorter 5′-UTRs defined by the TSSs identified in this research, we searched the −10 motifs within 0-35 bp upstream of TSSs. Many of the 215 putative sigma 38–dependent genes were known to be part of the sigma 38 regulon, e.g., *osmC*, *frdA*, *cfa*, *dps*, *galP*, *ldcC*, *proP*, *yohF*, *blc*, *yjgB*, *ygaU*, etc. [37]. Therefore, it is possible that any newly identified genes in this subset encode proteins that play active roles in *Salmonella* survival during stationary phase or stress responses. In addition to sigma 38 regulons, we also identified 863 possible sigma 70 regulons. The promoter regions for 172 of these genes had clear −35 and −10 motifs. For the remainder of genes, the −35 motifs were not identified, most likely because of significant degeneration at this site [14]. These putative sigma 70-dependent genes are likely important for normal *S.* Typhimurium growth.

## Conclusions

We have developed an empirical method to detect bacterial TSSs and TTSs from directional RNA-seq data, demonstrating that this type of data can be used to identify transcriptional borders in bacteria. The method proved to be more accurate than other similar applications and achieved a maximum resolution of ±10-20 nt. We used our method to identify numerous TSSs and TTSs within the *S*. Typhimurium 14028s genome and generated a curated inventory of 14028s operons and sigma factor regulons. These databases will benefit the *Salmonella* research community. The methods and observations that we have described can also be applied to directional RNA-seq data obtained from diverse bacterial species to detect transcript structure automatically and, in general, to enhance the study of bacterial transcriptomes.

### Availability

The software tools as well as the manuals can be freely downloaded with the link: http://www.vido.org/bactranscriptstructure (Username: BMC Genomics).

## Methods

### RNA-seq datasets

cDNA libraries were generated from mRNA pools purified from *S.* Typhimurium 14028s. The total of 54 libraries consisted of: 12 libraries prepared from 14028s planktonic cells, isolated at four different growth phases; 9 libraries prepared from 14028s multicellular biofilm aggregates at three different growth phases; 12 libraries prepared from planktonic cells of a biofilm-deficient mutant (Δ*csgD*;[38]) at four growth phases; and 21 libraries (12 from planktonic cells, 9 from multicellular aggregates) prepared from a *S.* Typhimurium 14028s reporter strain containing a *csgD* promoter::*luxCDABE* fusion plasmid [38]. The specific details about the RNA extraction and library preparation protocols is described elsewhere [39]. All sequencing was performed on the Illumina HiSeq 2000. The raw reads from each library were mapped to *S*. Typhimurium 14028s genome with Geneious v. 5.6.5 (www.biomatters.com).

Three *E. coli* GAII unpaired-end RNA-seq libraries were downloaded from http://bioinfolab.uncc.edu/TruHmm_package/raw_data/Ecoli_raw_reads/ (‘LB.GAII.fastq.gz’, ‘HS15min_r3.GAII.fastq.gz’ and ‘M-P4h_r3.GAII.fastq.gz’) [6]. The details about sample processing, RNA isolation, purification, amplification and sequencing are described in the original report [6]. The raw reads were mapped to the *E. coli* K-12 genome with the similar parameter settings as for the *S.* Typhimurium RNA-seq analysis stated above.

### Empirical and model-based methods for identification of TSSs and TTSs

To calculate the read coverage for each annotated protein-encoding gene, the depth at each genomic position was counted. Genes were excluded from empirical TSS and TTS detection based on the following three criteria: (1) < 70% whole coding region coverage, (2) average per-site read depth < 3 along the coding regions, or (3) <10% coverage at 5′ ends (for TSSs) or 3′ ends (for TTSs). Remaining genes were analyzed with a single-nucleotide extension strategy beginning from the start codon and moving upstream for TSS or from the stop codon and moving downstream for TTS. For each nucleotide extension, the read depths at that position and the neighboring position (upstream for TSS and downstream for TTS) were compared using the identification standards shown in Figure 1, until the borders were identified or not found after 200 iterations.

The Poisson distribution-based model (McClure method) was implemented according to [7] and compared with our empirical method. The McClure method makes the assumption that reads are randomly sampled within a gene region, including the untranslated regions for a protein-encoding gene [7]. Identifying the TSS and TTS of a gene is identical to determining the boundaries at which the gene starts to be transcribed while the distantly adjacent genomic regions are not transcribed or transcribed within neighbor genes. Repeated single nucleotide extension to a prior gene region is performed to determine the boundaries. Assume a protein-encoding gene, and the coding region *G* represents the prior gene region. The probability of the read number sampled within the region *G* can be fit with a Poisson distribution, $\Pr \left(k\Big|G\right)=\frac{\lambda^k\cdot {e}^{-\lambda }}{k!},$ where *k* represents the number of reads and the parameter *λ* can be easily estimated according to the distribution of reads within *G*. Similarly, for a background (*B*) read distribution, the probability Pr(*k*|*B*) can also be calculated with the similar formula of Pr(*k*|*G*) but the *λ* should be replaced with the average background read number. The antisense-strand regions throughout the whole genome could be considered as the background. A *G*-adjacent position *s* (e.g., 5′-side) could either belong to *G* (*G*|*s*) or a non-transcribed region *N* (*N*|*s*). The only information for boundary discrimination is read number so that the probability of Pr(*G*|*s*) and Pr(*N*|*s*) are transformed to Pr(*G*|*x*) and Pr(*N*|*x*), respectively, where *x* represents the read number at position *s*. Based on Bayes’ theorem, Pr(*G*|*x*) and Pr(*N*|*x*) can be calculated as:

$\Pr \left(C\kern0.1em \Big|x\right)=\frac{ \Pr (C)\cdot \Pr \left(x\;\Big|C\right)}{ \Pr (x)},$ where *C* represents *G* or *B*. According to Bayesian classification criteria and the assumed equal Pr(*C*) for transcribed gene region or background non-transcribed region, the class (*G* or *B*) with the maximal likelihood Pr(*x*|*C*) should also have the maximal posteriori probability and therefore have the maximal probability to be true. The *G* region is extended by this way, and the *λ* value for *G* is also updated recursively.

### Read-based and library-based statistical models

A binomial distribution B_r_(n_r_, p_r_) was modeled for testing the significance of the read depth at the border site rather than the adjacent position, where n_r_ is the total number of reads mapped to the TSSs/TTSs border and adjacent sites for the target gene, and p_r_ is ½, representing the probability of a random distribution. Another binomial distribution B_l_(n_l_, p_l_) was also modeled to calculate the random probability of repeat of the same TSSs/TTSs detected among different libraries. Let n_0_ represent the number of possible TSSs or TTSs detected for a target gene. In this distribution model, n_l_ represents the number of libraries, while p_l_ is the inversion of (n_0_ + 1). The *p* values obtained with the models were further corrected by the False Discovery Rate (FDR), with significance level set as FDR < 0.05.

### Analysis of operon organization

The pipeline for operon organization analysis was shown in Figure 6A, and is described here briefly. The intergenic length was calculated for each pair of annotated genes in *S*. typhimurium 14028s. A cutoff of an intergenic distance of 200 bp between adjacent genes was set to discern orphan operons. For the single genes with a <200 bp intergenic length at one or two ends, if both TSS and TTS were defined (or with a ≥200 bp intergenic length at one end and a defined transcription border at the other end which showed a <200 bp intergenic length), orphan-like operons were defined. For potential multiple-gene operons, the expression correlation coefficients (Pearson Correlation Coefficients, PCCs) were calculated. If the neighbor genes were highly expressed under some conditions, and PCC ≥ 0.5, they were considered to belong to a unique operon with co-expression validation; if the neighbor genes were highly expressed under some conditions, but PCC < 0.5, they were considered to belong to two neighboring operons; if one or both of the neighbor genes was not or lowly expressed under any condition, the genes were still considered within a unique operon inferred by intergenic distance. The multiple operons were obtained based on the relationship between two neighbor genes and a process of iterative extensions. Some single genes separated from the multiple-gene operons by co-expression analysis were re-classified into the orphan-like category. For co-expression analysis, the read number for each gene in each library was normalized, and then a logarithmic transformation was performed.

### Detection of sigma factor binding motifs in promoter

For each gene with a TSS identified, the 35 bp DNA sequence upstream of the TSS was extracted. For a gene that had more than one TSS identified (e.g., from other libraries), only the TSS furthest away from the start codon was used for analysis. A pattern ‘CTA[CT][ATG][CTA]T’ was searched in the 35 bp sequences for putative sigma 38 binding sites, and ‘[ATG]TA[AGCT][ACTG][CGTA]T’ was searched for −10 sigma 70 binding sites. A space between 10–25 nucleotides was allowed between two motifs that were used to detect the putative −35 sigma 70 binding motifs. The captured patterns were further shown for the nucleotide composition at each position with WebLogo [40].

### Availability of supporting data

RNA-seq data analyzed and used to develop the methods described are available in the SRA database (study #SRP056892; accession numbers: SRX976427, SRX976344, SRX976443, SRX976341, SRX976337, SRX976336, SRX976335, SRX974437, SRX976482, SRX976480, SRX976478, SRX976476, SRX976475, SRX976474, SRX976473, SRX976471, SRX976470, SRX976469, SRX976468).

## Additional files

Additional file 1:
**Transcriptional start sites and termination sites identified for chromosomally-encoded genes of** ***S.*** **Typhimurium 14028s.** Description of data: A complete list of all TSS/TTS in the *S.* Typhimurium 14028s genome as determined from analysis of RNAseq transcriptome data, with FDR corrected *p* values <0.05. Gene names and nucleotide positions are listed as annotated in the *S.* Typhimurium 14028s genome ([41]; NC_016856). TSS_Region: Ingenic - the predicted site lies within the coding region of an annotated gene; Intergenic - the predicted site lies between the coding sequences of two adjacent genes. Additional file 2:
**TSSs and TTSs predicted from** ***E. coli*** **GAII RNA-seq data.** Description of data: Worksheet 1 (E.Coli_GAII-TSS) – All TSSs identified from *E. coli* GAII datasets with the empirical method. Worksheet 2 (E.Coli_GAII-TSS) – All TTSs identified from *E. coli* GAII datasets with the empirical method. Worksheet 3 (Predicted vs. confirmed TSSs) – Distance between all predicted TSSs and known TSSs. Worksheet 4 (Sig_Pred vs. confirmed TSSs)) – Distance between significant predicted TSSs, with read number based statistical tests, and known TSSs. Additional file 3:
**Operon list for chromosomally-encoded genes of** ***S.*** **Typhimurium 14028s.** Description of data: Operon Start-End: nucleotide positions as listed in the *S.* Typhimurium 14028s genome (NC_016856). Gene(s): Gene names as listed in the annotated genome record. Operon-type: Listed as described in Figure 6. Annotation: Description of how the operons were defined – as described in the Results section of the main text. Additional file 4:
**List of** ***S.*** **Typhimurium 14028s genes with promoter regions containing sigma 38 or sigma 70 binding motifs.** Description of data: Worksheet 1 – genes where the −10 promoter region has a sigma 38 binding motif. Worksheet 2 – genes where the −10 promoter region has a sigma 70 binding motif. Worksheet 3 – genes where the −35 promoter region has a sigma 70 binding motif.
